# ‘Red blood transfusion in patients undergoing cardiac surgery reply’

**DOI:** 10.1007/s12471-015-0695-4

**Published:** 2015-05-21

**Authors:** M.C. Haanschoten, A.H.M. van Straten, M.A. Soliman Hamad

**Affiliations:** 1 Department of Anesthesiology, Department of Intensive Care Unit, Catharina Hospital, Eindhoven, The Netherlands; 3 Department of Cardiothoracic Surgery, Catharina Hospital, Michelangelolaan 2, PO Box 1350, 5602 ZA Eindhoven, The Netherlands

To the editor,

We would like to thank Dr. Noyez for his comments [1] on our study [2]. In our study, we investigated the safety and logistic convenience of the No Elective Red Cells (NERC) program. We presented the results of the first 500 patients who were managed according to this strategy. According to these results, it was safe to perform cardiac surgery without the immediate availability of blood in the operating room. Transfusion was avoided in 81 % of the patients. Predictors for perioperative blood transfusion were female, left ventricular function and EuroSCORE.

An important question that was raised in Dr. Noyez’s comment [1] is about the percentage of red cell units returned to the blood bank and the percentage of units that could no longer be used because of reduced quality. This was not one of our endpoints when designing the study. We did not have a control group, which is one of the limitations of this study. However, the question could be answered in a new study design apart from the NERC program. It is possible to review the database of the blood bank in a certain period in order to find an answer to this relevant question.

One of the advantages of this new strategy is the possibility to decrease the number of units of blood in the stock of the hospital blood bank. As a result, the number of units of blood supplied by the central Dutch blood bank to our blood bank has decreased. This leads to less general requests for blood donation and consequently to a decrease in the costs.

In our study [2], we have shown that blood was available within 20 min of ordering it for all patients who needed blood transfusion intraoperatively. Without this logistic convenience, this program would not have been considered successful.

In our series of 500 patients, we did not encounter any catastrophic bleeding problems that necessitated emergency blood transfusion. We agree with the author that these problems can occur even in the simplest procedure. In case of an emergency, blood units can be delivered within 10 min of ordering. This is facilitated by preoperative typing and screening of all patients.
